# Associations Between Late‐Night Shift Work and Perinatal Outcomes: A Nationwide Cross‐Sectional Study Using JACSIS and JASTIS Data

**DOI:** 10.1111/jog.70205

**Published:** 2026-02-13

**Authors:** Yuya Tanaka, Yoshifumi Kasuga, Yoshihiko Hosokawa, Junko Tamai, Yuka Fukuma, Toshimitsu Otani, Marie Fukutake, Satoru Ikenoue, Takahiro Tabuchi, Mamoru Tanaka

**Affiliations:** ^1^ Department of Obstetrics and Gynecology Keio University School of Medicine Shinjuku Tokyo Japan; ^2^ Department of Obstetrics and Gynecology Miyazaki Prefectural Miyazaki Hospital Miyazaki Miyazaki Japan; ^3^ Division of Epidemiology, School of Public Health Tohoku University Graduate School of Medicine Sendai Miyagi Japan

**Keywords:** late‐night shift, nationwide cross‐sectional study, perinatal outcome, pregnancy, work

## Abstract

**Aim:**

This study investigated associations between late‐night shift work and perinatal outcomes.

**Methods:**

Participants were pregnant women with recorded perinatal outcomes (late‐night shift group, 626 cases; non‐late‐night shift group, 6633 cases) identified from two Japanese Internet surveys. We analyzed the association between late‐night shift work and adverse perinatal outcomes. Furthermore, we examined factors associated with the use of Maternal Health Management and Guidance Cards.

**Results:**

The late‐night shift group had significantly higher rates of threatened miscarriage, premature rupture of membranes (PROM), use of the Maternal Health Management and Guidance Card, health problems requiring hospitalization, fetal health problems, and infectious diseases compared with the non‐late‐night shift group. However, other perinatal outcomes, including preterm birth, gestational age at delivery, mode of delivery, and birth weight, did not differ significantly between groups. Among late‐night shift‐working mothers, those who used the Maternal Health Management and Guidance Card had a higher incidence of hyperemesis gravidarum, threatened miscarriage, and preterm labor than those who did not.

**Conclusions:**

Late‐night shift work during pregnancy may be associated with an increased risk of perinatal complications. The Maternal Health Management and Guidance Card may provide support for pregnant women engaged in late‐night shift work.

## 1 Introduction

With the growing participation of women in Japan's workforce, providing health support for pregnant employees has become critical. Following the enactment of the Act on Promotion of Women's Participation and Advancement in the Workplace in 2016, the number of female workers increased by approximately 3.7 million between 2012 and 2022 (Japanese data). Consistent with this trend, the prevalence of late‐night shift work among Japanese employees increased from 13.3% in 1997 to 17.8% in 2002, 17.9% in 2007, and 21.8% in 2012 (Japanese data). Consequently, the absolute number of pregnant workers engaged in late‐night shift work also increased. In Japan, the Labor Standards Act restricts employment beginning 6 weeks before the expected date of delivery (i.e., at 34 gestational weeks) [1]. Therefore, many pregnant workers, including those assigned to late‐night shifts, often continue working under unchanged conditions during pregnancy, especially during the first trimester when occupational adjustments are less likely.

Late‐night shift work is known to induce systemic and chronic stress by disrupting circadian rhythms and sleep patterns, resulting in widespread health effects [2]. Previous studies on nonpregnant workers have reported that such disruptions are associated with an increased risk of insomnia, gastrointestinal symptoms, metabolic syndrome, and cardiovascular diseases [2, 3, 4]. In 2007, the International Agency for Research on Cancer classified shift work involving circadian disruption as a probable human carcinogen (Group 2A) [5]. In addition, among pregnant women, working late‐night shifts is a risk factor for miscarriage [6]. Furthermore, shift work, including late‐night shifts, has been associated with miscarriage, preterm birth (PB: gestational age at delivery < 37 weeks), and low birth weight (LBW: birth weight < 2500 g) [7, 8]. However, a systematic review and meta‐analysis assessed the quality of this evidence as low to moderate [7]. A meta‐analysis examining the association between shift work, long working hours, and PB found no significant relationships [9]. These findings suggest the need for further investigation as the impact of late‐night shift work during pregnancy and perinatal outcomes remains unclear.

Additionally, in Japan, the Maternal Health Management and Guidance Card (Figure S1) is designed to support pregnant workers by facilitating appropriate work adjustments and health management during pregnancy. However, few studies have examined awareness and actual use of this card among pregnant women in Japan, and none have been published in English. Investigating awareness and use of this card may provide insights into early physical discomfort experienced by pregnant workers before the onset of complications.

Therefore, we investigated the association between late‐night shift work during pregnancy and perinatal outcomes. We also aimed to clarify pregnant women's awareness and actual use of the Maternal Health Management and Guidance Card.

## 2 Methods

### 2.1 Study Design, Data Setting, and Participants

In this nationwide, cross‐sectional study, we analyzed data from pregnant women with available perinatal outcomes obtained from the Japan COVID‐19 and Society Internet Survey (JACSIS; https://jacsis‐study.jp/) conducted between July and August 2021, and the Japan Society and New Tobacco Internet Survey (JASTIS; https://jastis‐study.jp/) conducted in February 2022. JACSIS and JASTIS collected data from pooled panels provided by an internet research agency (Rakuten Insight Inc.; https://insight.rakuten.co.jp/), which currently has approximately 2.3 million registered panelists. These panels comprise individuals aged 15–79 years, including pregnant and postpartum women as well as fetus–infant pairs. Details regarding JACSIS and JASTIS, including quality control procedures and policies for panelists, have been described elsewhere [10, 11, 12]. Participants in this study were therefore categorized into two groups: a late‐night shift group (*n* = 626) and a non‐late‐night shift group (*n* = 6633).

### 2.2 Definitions of Late‐Night Shifts and Perinatal Outcomes

The survey was designed to ask participants the question, Were you engaged in late‐night work (10:00 pm to 5:00 am) during pregnancy? Participants responded with “yes” or “no.” Accordingly, participants who worked during late‐night hours were classified into the late‐night shift group, whereas those who did not were classified into the non‐late‐night shift group. Perinatal outcomes, including the diagnosis of hypertensive disorders of pregnancy (HDP), were determined based on whether the participant had a history of hypertension or recorded a systolic blood pressure of ≥ 140 mmHg or diastolic blood pressure ≥ 90 mmHg during pregnancy [13], as documented in the Maternal and Child Health Handbook [10]. Similarly, participants used their Maternal and Child Health Handbook to provide information on gestational age at delivery and mode of delivery (vaginal or cesarean). Gestational diabetes mellitus (GDM) and cesarean section (CS) were self‐reported by participants.

### 2.3 Description of the Maternal Health Management and Guidance Card

Previous studies have suggested that appropriate adjustments to work tasks may help prevent perinatal complications and physical symptoms in pregnant workers [14]. In Japan, the Maternal Health Management and Guidance Card (Figure S1), issued by the Ministry of Health, Labour and Welfare, has been implemented since 1997 to help antepartum and postpartum workers balance health management with employment. The card serves as a tool for pregnant and postpartum workers to communicate doctors' medical instructions to their employers [15]. It was introduced as part of the maternal protection measures under the Labor Standards Act of Japan. Doctors can issue guidance tailored to the specific physical symptoms of pregnant women. These measures may include standard recommendations, such as taking leave, shortening working hours, and restricting certain tasks, as well as specific guidance, such as avoiding late‐night shifts, depending on the individual's condition. The use of the Maternal Health Management and Guidance Card facilitates appropriate communication on maternal health among doctors, pregnant workers, and employers, and is expected to contribute to creating safe and healthy working environments.

In this study, participants were asked whether they were aware of the card and whether they had used it to request work adjustments. Given the missing responses from some participants, analyses of recognition and use of the Maternal Health Management and Guidance Card were conducted using a subset of the total sample (*n* = 625 in the late‐night shift group and *n* = 5145 in the non‐late‐night shift group). Furthermore, we evaluated the association between the use of this card and perinatal outcomes in the late‐night shift group (*n* = 625) after adjusting for potential confounders.

### 2.4 Outcomes

The primary outcome was the association between late‐night shift work and perinatal outcomes. Subsequently, we analyzed the awareness of Maternal Health Management and Guidance Card and the reasons for its use. Perinatal complications included the following: worsening of pre‐existing medical conditions before pregnancy; hyperemesis gravidarum requiring hospitalization; HDP; gestational proteinuria (≥ 2+); GDM; threatened miscarriage requiring hospitalization or accompanied by abnormal vaginal bleeding; preterm labor requiring hospitalization; placenta previa or low‐lying placenta; placental abruption; premature rupture of membranes (PROM); other health problems requiring hospitalization; fetal health problems; infections other than the common cold; CS rate; and PB rate. In this study, “fetal health problems” may have included abnormalities or complications directly affecting the fetus, such as growth restriction or structural anomalies. “Health problems requiring hospitalization” may have included minor maternal health conditions that did not meet the criteria for obstetric complications but resulted in hospitalization or work leave due to worsening pregnancy‐related symptoms such as nausea, back pain, or fatigue.

### 2.5 Statistical Analysis

We conducted a logistic regression analysis for categorical variables to evaluate the association between late‐night shifts and perinatal outcomes, estimating odds ratios (ORs) and 95% confidence intervals (CIs). For continuous variables, we performed multiple linear regression analyses to estimate the unstandardized regression coefficient (B) and its 95% CI, as well as the standardized regression coefficient (*β*). The reference group was the non‐late‐night shift group. Associations between Maternal Health Management and Guidance Card use and perinatal outcomes were also examined using logistic and linear regression analyses, adjusting for the same set of covariates. Nonusers of the card served as the reference group.

The following confounding variables were included: maternal age at survey (< 35 years or ≥ 35 years); prepregnancy body mass index (BMI); parity (primipara or multipara); mode of conception (spontaneous conception or in vitro fertilization); education level (≤ 12 years [high school] or ≥ 13 years [university]); and smoking during pregnancy (yes: smoked at least once during pregnancy; no: never smoked). Analyses were conducted using a complete‐case approach, excluding participants with missing data for any of the variables included in the regression models.

Given the exploratory nature of the study and the multiple outcomes assessed, no correction for multiple testing was applied. Results should therefore be interpreted with caution, as hypothesis‐generating rather than confirmatory. Because the study was based on Internet panel data, no weighting adjustments were applied. Therefore, findings should be interpreted with caution regarding their generalizability to the overall pregnant population. All statistical analyses were performed using SPSS version 29.0.2.0 (IBM Corp., Armonk, NY, USA). Statistical significance was defined as a two‐sided *p* value < 0.05.

### 2.6 Ethics Statement

This study was approved by our institutional ethics committee (Approval No. 20241175), the Ethics Committee of Osaka International Cancer Institute (Approval No. 20084–12), and the Ethics Committee of Tohoku University Graduate School of Medicine (Approval No. 2024–1‐1035). All procedures were conducted in accordance with the ethical guidelines for medical and health research involving human subjects issued by the Japanese Ministry of Health, Labour and Welfare, and with the 1964 Helsinki Declaration and its later amendments. Informed consent was obtained electronically, and all participants were informed of their right to withdraw from the study.

## 3 Results

**FIGURE 1:**
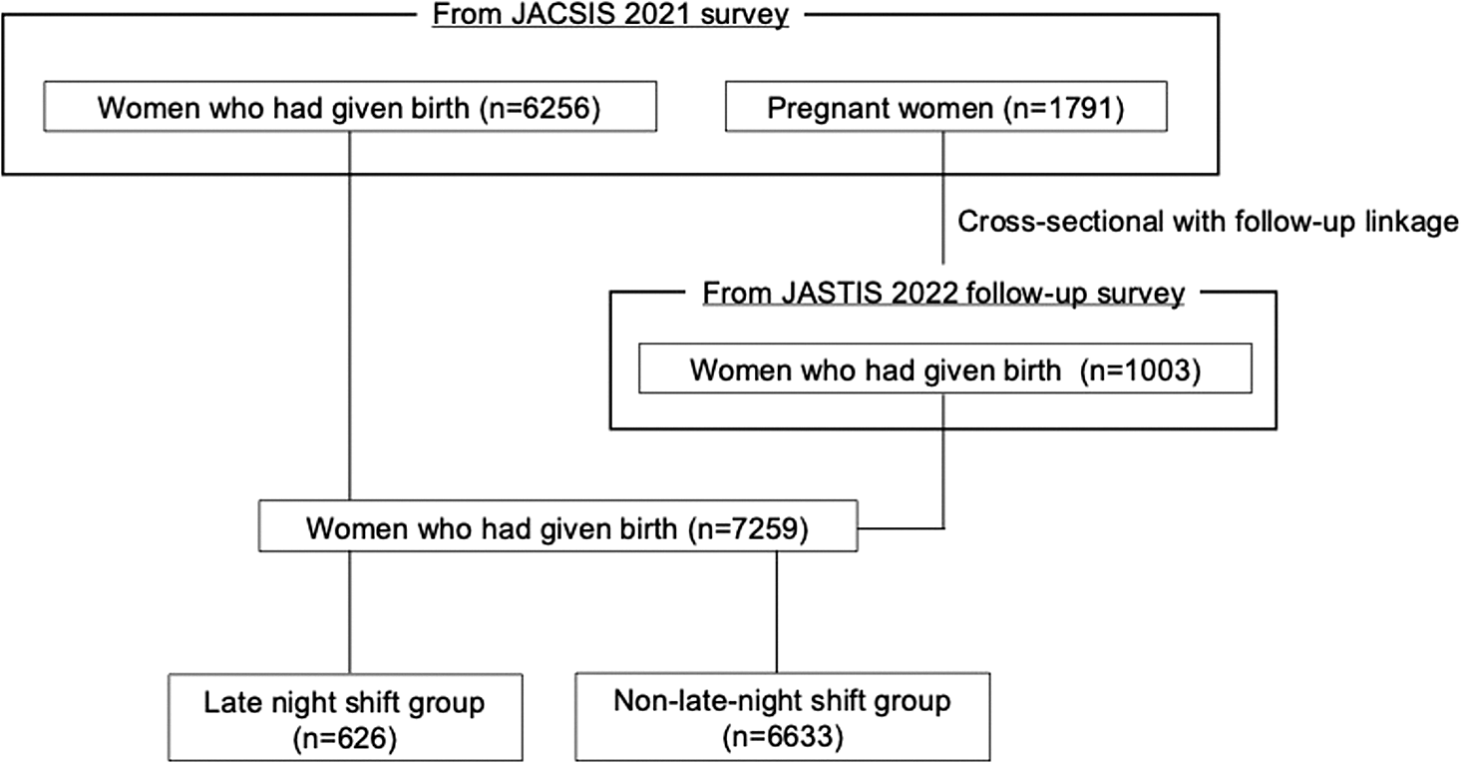
Flow chart of study participants. Participant data were obtained from the JACSIS 2021 survey and the JASTIS 2022 follow‐up survey in Japan. A total of 7259 women who had given birth were included in the analysis. These participants were categorized into two groups based on the presence or absence of late‐night shift work during pregnancy: The late‐night shift group (*n* = 626) and the non‐late‐night shift group (*n* = 6633).

Figure 1 shows the flow chart of study participants. Among the 8047 participants in the JACSIS 2021 survey conducted from July to August 2021, 6256 women (77.7%) had given birth. Among the remaining 1791 pregnant women (22.3%), 1003 (56.0%) who had given birth by the JASTIS 2022 follow‐up survey in February 2022 were included. Consequently, the total number of women who had given birth increased to 7259 (90.2%). Among them, 626 women (8.6%) were classified into the late‐night shift group and 6633 (91.4%) into the non‐late‐night shift group.

The characteristics of the participants included in this analysis are summarized in Table 1. Maternal age at survey, nulliparity, and smoking during pregnancy showed highly significant differences (*p* < 0.001), and educational attainment of ≥ 13 years also differed significantly between the two groups (*p* = 0.017). In contrast, prepregnancy BMI was comparable between the two groups. Logistic regression analysis revealed that the prevalence of threatened miscarriage requiring hospitalization or accompanied by abnormal vaginal bleeding was 5.8% in the late‐night shift group and 3.8% in the non‐late‐night shift group, with a significantly higher prevalence in the late‐night shift group (OR = 1.56, 95% CI, 1.09–2.24, *p* = 0.016). The prevalence of PROM was significantly higher in the late‐night shift group (9.7%) than in the non‐late‐night shift group (6.2%) (OR = 1.48, 95% CI, 1.11–1.97, *p* = 0.007).

**TABLE 1:** Characteristics of participants by late‐night shift work status.

	Mean ± SD or N (%)			
	All pregnant women (*n* = 7259)	Late‐night shift (*n* = 626)	Non‐late‐night shift (*n* = 6633)	*p*
Maternal age at survey, years	32.1 ± 4.4	31.0 ± 4.5	32.2 ± 4.4	< 0.001 *
≥ 35 years, *n* (%)	2132 (29.4)	141 (22.5)	1991 (30.0)	
Prepregnancy BMI, kg/m^2^	23.3 ± 19.7	23.5 ± 18.6	23.3 ± 19.8	0.78
BMI category				0.50
< 18.5, *n* (%)	1366 (18.8)	110 (17.6)	1256 (18.9)	
18.5–24.9, *n* (%)	5163 (71.1)	458 (73.2)	4705 (70.9)	
≥ 25, *n* (%)	730 (10.1)	58 (9.3)	672 (10.1)	
Nulliparity, *n* (%)	4231 (58.3)	429 (68.5)	3802 (57.3)	< 0.001 *
In vitro fertilization, *n* (%)	549 (7.6)	42 (6.7)	507 (7.6)	0.43
Smoking during pregnancy, *n* (%)	331 (4.6)	48 (7.7)	283 (4.3)	< 0.001 *
Educational attainment ≥ 13 years, *n* (%)	6075 (83.7)	545 (87.1)	5530 (83.4)	0.017 *

Abbreviations: BMI, body mass index; SD, standard deviation.

* Statistically significant differences between groups are displayed. Data are mean ± SD and *n* (%).

Similarly, the prevalence of other health problems requiring hospitalization was 5.8% and 3.5% (OR = 1.67, 95% CI, 1.16–2.40, *p* = 0.006), fetal health problems were 5.1% and 3.4% (OR = 1.52, 95% CI, 1.04–2.23, *p* = 0.031), and infections other than the common cold were 4.2% versus 2.5% (OR = 1.64, 95% CI, 1.07–2.51, *p* = 0.023), all of which were significantly higher in the late‐night shift group. However, no significant differences were observed in the CS and PB rates between the two groups (Table 2). Linear regression analysis showed no significant differences in either birth weight or gestational age at delivery between the two groups (Table 3).

**TABLE 2:** Perinatal outcomes associated with late‐night shift work.

	Late‐night shift (*n* = 626)	Non‐late‐night shift (*n* = 6633)	*p*	ORs	95% CI
Worsening of pre‐existing medical conditions before pregnancy	20 (3.2)	177 (2.7)	0.62	1.13	0.70–1.81
Hyperemesis gravidarum requiring hospitalization	32 (5.1)	229 (3.5)	0.077	1.43	0.99–2.10
Hypertensive disorders of pregnancy (HDP)^a^	34 (5.4)	336 (5.1)	0.70	1.07	0.74–1.55
Gestational proteinuria (≥ 2+ proteinuria)	52 (8.3)	475 (7.2)	0.38	1.15	0.85–1.56
Gestational diabetes mellitus	26 (4.2)	375 (5.7)	0.20	0.76	0.51–1.15
Threatened miscarriage requiring hospitalization or accompanied by hemorrhage	36 (5.8)	251 (3.8)	0.016*	1.56	1.10–2.24
Preterm labor requiring hospitalization	41 (6.5)	425 (6.4)	0.95	1.01	0.72–1.41
Placenta previa or low‐lying placenta	11 (1.8)	214 (3.2)	0.051	0.54	0.29–1.00
Placental abruption	3 (0.5)	41 (0.6)	0.64	0.77	0.23–2.46
Premature rupture of membranes (PROM)	61 (9.7)	413 (6.2)	0.007*	1.48	1.11–1.97
Other health problems requiring hospitalization^b^	36 (5.8)	232 (3.5)	0.006*	1.68	1.17–2.40
Fetal health problems^c^	32 (5.1)	226 (3.4)	0.031*	1.52	1.04–2.23
Physician‐diagnosed infections other than the common cold	26 (4.2)	169 (2.5)	0.023*	1.64	1.07–2.51
Mode of delivery (cesarean section rates)	107 (17.1)	1290 (19.4)	0.28	0.90	0.71–1.10
Preterm birth	38 (6.1)	381 (5.7)	0.90	1.03	0.73–1.45

Abbreviations: CI, confidence interval; ORs, odds ratio.

* Statistically significant differences between groups are dysplayed. Data are presented as *n* (%).

^a^ The diagnosis of hypertensive disorders of pregnancy (HDP) was based on whether the participant had a history of hypertension, systolic blood pressure of ≥ 140 mmHg, or a diastolic blood pressure of ≥ 90 mmHg during pregnancy.

^b^ “Other health problems requiring hospitalization” may have included minor maternal health conditions that did not meet the criteria for obstetric complications but required hospitalization or work leave due to worsening pregnancy‐related symptoms (e.g., nausea, back pain, or fatigue).

^c^ “Fetal health problems” may have included abnormalities or complications directly affecting the fetus, such as growth restriction or structural anomalies.

**TABLE 3:** Gestational age at delivery and birth weight associated with late‐night shift work.

	Late‐night shift (*n* = 626)	Non‐late‐night shift (*n* = 6633)	*p*	*B*	95% CI	*β*
Gestational age at delivery (weeks)	39.2 ± 2.03	39.1 ± 1.90	0.34	0.080	−0.084 ~ 0.25	0.012
Birth weight (g)	3010.5 ± 429.2	3005.1 ± 419.6	0.70	7.19	−28.99 ~ 43.21	0.005

*Note:* Data are mean ± SD.

Abbreviations: *B*, unstandardized regression coefficient; CI, confidence interval; SD, standard deviation; *β*, standardized regression coefficient.

We also analyzed the recognition and use of Maternal Health Management and Guidance Cards (Table 4). Awareness of the Maternal Health Management and Guidance Card was 69.8% in the late‐night shift group (*n* = 625) and 57.3% in the non‐late‐night shift group (*n* = 5145), with a significantly higher prevalence in the late‐night shift group (OR = 1.73, 95% CI, 1.44–2.07, *p* < 0.001). Likewise, card use was 19.5% in the late‐night shift group and 11.3% in the non‐late‐night shift group, again significantly higher in the late‐night shift group (OR = 1.92, 95% CI, 1.54–2.38, *p* < 0.001).

**TABLE 4:** Recognition and use of the maternal health management and guidance card associated with late‐night shift work.

	Late‐night shift (*n* = 625)	Non‐late‐night shift (*n* = 5145)	*p*	ORs	95% CI
Recognizing the “Maternal Health Management and Guidance Card”	436 (69.8)	2950 (57.3)	< 0.001 *	1.73	1.44–2.07
Use of the “Maternal Health Management and Guidance Card”	122 (19.5)	580 (11.3)	< 0.001 *	1.92	1.54–2.38

Abbreviations: CI, confidence interval; ORs, odds ratio.

* Statistically significant differeces between groups are dysplayed. Data are presented as *n* (%).

Furthermore, we evaluated the association between use of the Maternal Health Management and Guidance Card and perinatal outcomes in the late‐night shift group (*n* = 625), adjusting for potential confounders. Logistic regression analysis showed that the prevalence of hyperemesis gravidarum requiring hospitalization was 12.3% among card users and 3.4% among card nonusers (OR = 4.28, 95% CI, 2.05–8.93, *p* < 0.001). Similarly, the prevalence of threatened miscarriage requiring hospitalization or accompanied by abnormal vaginal bleeding was 13.9% versus 3.8% (OR = 4.56, 95% CI, 2.25–9.25, *p* < 0.001), and preterm labor requiring hospitalization was 13.9% versus 4.8% (OR = 3.14, 95% CI, 1.62–6.11, *p* < 0.001), both significantly higher among card users.

However, no significant differences were observed in the prevalence of PROM, other health problems requiring hospitalization, fetal health problems, or physician‐diagnosed infections other than the common cold between card users and nonusers (Table 5). Finally, the linear regression analysis showed a statistically significant difference in gestational age at delivery between card users and nonusers (*p* = 0.030), whereas no significant difference was observed in birth weight (*p* = 0.73) (Table 6).

**TABLE 5:** Perinatal outcomes associated with the use of the maternal health management and guidance card in the late‐night shift group.

	Card users (*n* = 122)	Card nonusers (*n* = 503)	*p*	ORs	95% CI
Worsening of pre‐existing medical conditions before pregnancy	4 (3.3)	16 (3.2)	0.92	1.06	0.35–3.27
Hyperemesis gravidarum requiring hospitalization	15 (12.3)	17 (3.4)	< 0.001 *	4.28	2.05–8.93
Hypertensive disorders of pregnancy (HDP)^a^	8 (6.6)	26 (5.2)	0.41	1.42	0.62–3.26
Gestational proteinuria (≥ 2 + proteinuria)	12 (9.8)	40 (8.0)	0.39	1.36	0.68–2.71
Gestational diabetes mellitus	3 (2.5)	23 (4.6)	0.33	0.54	0.16–1.86
Threatened miscarriage requiring hospitalization or accompanied by hemorrhage	17 (13.9)	19 (3.8)	< 0.001 *	4.56	2.25–9.25
Preterm labor requiring hospitalization	17 (13.9)	24 (4.8)	< 0.001 *	3.14	1.62–6.11
Placenta previa or low‐lying placenta	3 (2.5)	8 (1.6)	0.64	1.39	0.34–5.67
Placental abruption	2 (1.6)	1 (0.2)	0.07	9.60	0.82–111.86
Premature rupture of membranes (PROM)	13 (10.7)	48 (9.5)	0.66	1.16	0.60–2.24
Other health problems requiring hospitalization^b^	11 (9.0)	25 (5.0)	0.06	2.06	0.97–4.36
Fetal health problems^c^	5 (4.1)	27 (5.4)	0.60	0.77	0.29–2.06
Physician‐diagnosed infections other than the common cold	6 (4.9)	20 (4.0)	0.61	1.27	0.49–3.28
Mode of delivery (cesarean section rates)	107 (17.1)	1290 (19.4)	0.52	1.19	0.71–2.00
Preterm birth	7 (5.7)	31 (6.2)	0.92	1.04	0.44–2.46

Abbreviations: CI, confidence interval; ORs, odds ratio.

* Statistically significant differences between groups are desplayed. Data are presented as *n* (%).

^a^ The diagnosis of hypertensive disorders of pregnancy (HDP) was based on whether the participant had a history of hypertension, systolic blood pressure of ≥ 140 mmHg, or a diastolic blood pressure of ≥ 90 mmHg during pregnancy.

^b^ “Other health problems requiring hospitalization” may have included minor maternal health conditions that did not meet the criteria for obstetric complications but required hospitalization or work leave due to worsening pregnancy‐related symptoms (e.g., nausea, back pain, or fatigue).

^c^ “Fetal health problems” may have included abnormalities or complications directly affecting the fetus, such as growth restriction or structural anomalies.

**TABLE 6:** Gestational age at delivery and birth weight associated with the use of the maternal health management and guidance card in the late‐night shift group.

	Card users (*n* = 122)	Card nonusers (*n* = 503)	*p*	*B*	95% CI	*β*
Gestational age at delivery (weeks)	38.9 ± 2.9	39.3 ± 1.7	0.030 *	−0.45	−0.85 to −0.043	−0.087
Birth weight (g)	2999.1 ± 415.9	3013.1 ± 433.2	0.734	−14.84	−100.73 to 71.05	−0.014

Abbreviations: *B*, unstandardized regression coefficient; CI, confidence interval; SD, standard deviation; *β*, standardized regression coefficient.

* Statistically significant differecens between groups are desplayed. Data are mean ± SD.

## 4 Discussion

This nationwide study of 7259 pregnant women provides important evidence on the association between late‐night shift work and perinatal outcomes, representing one of the largest investigations of this issue in Japan. The findings revealed that late‐night shift work was associated with several adverse perinatal outcomes, but not PB and birth weight. Late‐night shift workers frequently requested Maternal Health Management and Guidance Cards due to perinatal complications.

Late‐night shift work is physically demanding and imposes a substantial physiological burden. It disrupts circadian rhythms and sleep patterns, thereby inducing systemic and chronic stress that has been linked to various health effects, including increased risks of insomnia, gastrointestinal symptoms, metabolic syndrome, and cardiovascular diseases [2, 3, 4]. In pregnant women, previous studies have suggested associations between late‐night shift work and adverse perinatal outcomes, including miscarriage, PB, HDP, and LBW [7, 16, 17, 18]. However, these studies were limited by their focus on specific occupations and relatively small sample sizes.

Although Kader et al. conducted a meta‐analysis, inconsistencies in the definitions and assessment methods of late‐night work across studies, combined with insufficient adjustment for confounders, produced inconsistent results [16]. Consequently, the quality of evidence was rated as low to moderate, and no definitive conclusions could be drawn. Our data revealed that some perinatal complications were more frequently detected in the late‐night shift group than in the non‐late‐night shift group, whereas no differences were observed for PB or birth weight. We analyzed the association between perinatal outcomes and use of the Maternal Health Management and Guidance Card to investigate this finding further. This card is part of a nationwide system for working pregnant women, allowing clinicians to provide written recommendations that are recognized across all clinics and facilities in Japan.

Uniform prohibition of late‐night shift work for pregnant workers is unnecessary. However, appropriate work adjustments should be made according to individual physical symptoms. This study revealed that pregnant women who worked late‐night shifts were more likely to use the card and request work adjustment. Particularly, pregnant women diagnosed with hyperemesis gravidarum, threatened miscarriage, or preterm labor were more likely to use the card to request workload reduction and other work adjustments. These findings suggest that the use of the Maternal Health Management and Guidance Card to facilitate flexible work modifications tailored to individual symptoms is a practical approach to mitigating the burden of late‐night work.

Although the current system functions to some extent, awareness of the Maternal Health Management and Guidance Card was only 69.6% in the late‐night shift group and 57% in the non‐late‐night shift group, indicating that many workers remained unaware of the system. Further dissemination and promotion of card use are necessary to ensure that its benefits reach a broader population of pregnant workers. Achieving this goal requires establishing workplace environments where pregnant workers feel comfortable discussing their workloads and health concerns. Clinicians should offer guidance tailored to pregnancy‐related symptoms and workload, while employers must actively seek to understand the health conditions of pregnant workers and implement flexible work adjustments. Through these efforts, collaboration among healthcare providers, pregnant workers, and employers will be essential for building an effective support system that utilizes the Maternal Health Management and Guidance Card.

A major strength of this study is its comprehensive evaluation of the association between late‐night shift work during pregnancy and perinatal outcomes, based on a nationwide internet‐based survey conducted with a unified definition of night work encompassing a wide range of occupations. However, there were some limitations. First, the gestational week at which pregnant women engaged in late‐night shift work, as well as the frequency or actual working hours of these shifts, were not considered because this study was a nationwide cross‐sectional study using an Internet‐based survey, and information on the timing of the late‐night shift was not collected. As the health status of pregnant women and the risk of perinatal complications change with gestational age, accounting for the timing of night shifts may help clarify the risks of specific perinatal complications. Further detailed investigations are needed to clarify the impact of night shift work, including a re‐evaluation of the definitions and assessment criteria for the frequency and working hours of late‐night shifts.

Second, although the overall sample size was large, the subgroup analyses—particularly those related to card users—were based on relatively small sample sizes. This limitation may have reduced the statistical power to detect associations within these subgroups. Third, perinatal complications were self‐reported by participants rather than verified by clinicians. Therefore, the evaluation of perinatal outcomes may be subject to several biases. Fourth, the cross‐sectional nature of the study made it impossible to evaluate whether the Maternal Health Management and Guidance Card reduced the risk of adverse perinatal outcomes. Further research is required to investigate whether the card can improve adverse perinatal outcomes.

In conclusion, late‐night shift work during pregnancy was associated with an increased risk of perinatal complications, though no associations were found with PB or birth weight. The Maternal Health Management and Guidance Card was frequently requested by pregnant women working late‐night shifts and may serve as a useful tool for facilitating work adjustments. Considering maternal and infant health and well‐being, pregnant workers engaged in late‐night shifts should be managed with support.

## Author Contributions

**Yuya Tanaka:** writing – original draft, writing – review and editing, investigation, conceptualization. **Junko Tamai:** investigation, writing – review and editing. **Yoshifumi Kasuga:** conceptualization, investigation, writing – review and editing.

## Funding

The authors have nothing to report.

## Consent

Informed consent was obtained from all participants at the time of participation in the original surveys.

## Conflicts of Interest

The authors declare no conflicts of interest. Dr. Yoshifumi Kasuga is an Editorial Board member of the submitted JOGR Journal and a co‐author of this article. To minimize bias, they were excluded from all editorial decision‐making related to the acceptance of this article for publication.

## Supporting information


**Figure S1:** The Maternal Health Management and Guidance Card.
The Maternal Health Management and Guidance Card (https://www.mhlw.go.jp/content/11900000/001066874.pdf, accessed on October 12, 2025), issued by the Ministry of Health, Labour and Welfare, has been implemented since 1997 to help antepartum and postpartum workers balance health management with employment. The card serves as a tool for pregnant and postpartum workers to communicate doctors' medical instructions to their employers.

## Data Availability

The data supporting the findings of this study were obtained from the “Japan COVID‐19 and Society Internet Survey (JACSIS)” and the “Japan Society and New Tobacco Internet Survey (JASTIS).” These datasets contain individual‐level information collected under informed consent and are subject to ethical and legal restrictions for the protection of personal information under the Ethical Guidelines for Medical and Health Research Involving Human Subjects in Japan. Therefore, the data are not publicly available. Access to the data is restricted and governed by the respective data management committees of the surveys.
