# Supplementary figures and images for: Associations Between Late‐Night Shift Work and Perinatal Outcomes: A Nationwide Cross‐Sectional Study Using JACSIS and JASTIS Data
Source: J Obstet Gynaecol Res. 2026 Feb 13;52(2):e70205. doi: 10.1111/jog.70205 (PMC12905467; doi:10.1111/jog.70205)

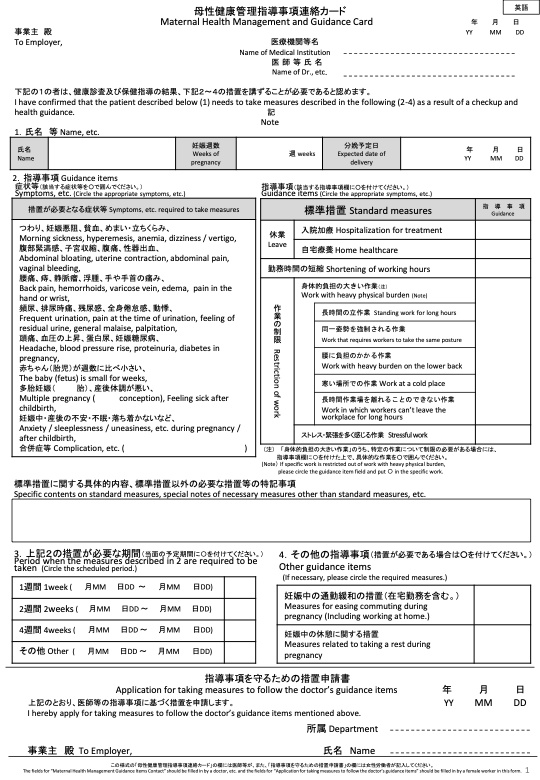

Supplement: Supplementary file 1 — Figure S1: The Maternal Health Management and Guidance Card. The Maternal Health Management and Guidance Card (https://www.mhlw.go.jp/content/11900000/001066874.pdf, accessed on October 12, 2025), issued by the Ministry of Health, Labour and Welfare, has been implemented since 1997 to help antepartum and postpartum workers balance health management with employment. The card serves as a tool for pregnant and postpartum workers to communicate doctors' medical instructions to their employers. [file JOG-52-0-s001.tiff]
